# A process and mechanism of action evaluation of the effect of early and intensive nutrition care, delivered via telephone or mobile application, on quality of life in people with upper gastrointestinal cancer: a study protocol

**DOI:** 10.1186/s12885-018-5089-8

**Published:** 2018-11-29

**Authors:** Kate Furness, Catherine E. Huggins, Lauren Hanna, Mary Anne Silvers, Paul Cashin, Liang Low, Daniel Croagh, Terry P. Haines

**Affiliations:** 1 Nutrition and Dietetics, Monash Health, Monash Medical Centre, 246 Clayton Road, Clayton, VIC 3168 Australia; 2 Department of Nutrition, Dietetics and Food, School of Clinical Sciences, Faculty of Medicine Nursing and Health Sciences, Monash University, Clayton, VIC 3168 Australia; 3 Department of Physiotherapy, School of Primary and Allied Health Care, Faculty of Medicine, Nursing and Health Sciences, Monash University, Frankston, VIC 3199 Australia; 4 Upper Gastrointestinal and Hepatobiliary Surgery, Monash Medical Centre, Clayton, VIC 3168 Australia; 5 Department of Surgery, School of Clinical Sciences, Faculty of Medicine, Nursing and Health Sciences, Monash University, Clayton, VIC 3168 Australia; 6 School of Primary and Allied Health Care, Faculty of Medicine, Nursing and Health Sciences, Monash University, Frankston, VIC 3199 Australia

**Keywords:** Process evaluation, mHealth, Behaviour change, Effectiveness, Engagement, Dietetic intervention, Oesophagogastric, Pancreatic cancer

## Abstract

**Background:**

Cancers of the upper gastrointestinal tract commonly result in malnutrition, which increases morbidity and mortality. Current nutrition best practice lacks a mechanism to provide early and intensive nutrition support to these patients. A 3-arm parallel randomised controlled trial is testing the provision of a tailored, nutritional counselling intervention delivered using a synchronous, telephone-based approach or an asynchronous, mobile application-based approach to address this problem. This protocol outlines the design and methods that will be used to undertake an evaluation of the implementation process, which is imperative for successful replication and dissemination.

**Methods:**

A concurrent triangulation mixed methods comparative analysis will be undertaken. The nutrition intervention will be provided using best practice behaviour change techniques and communicated either via telephone or via mHealth. The implementation outcomes that will be measured are: fidelity to the nutrition intervention protocol and to the delivery approach; engagement; acceptability and contextual factors. Qualitative data from recorded telephone consultations and written messages will be analysed through a coding matrix against the behaviour change techniques outlined in the standard operating procedure, and also thematically to determine barriers and enablers. Negative binomial regression will be used to test for predictive relationships between intervention components with health-related quality of life and nutrition outcomes. Post-intervention interviews with participants and health professionals will be thematically analysed to determine the acceptability of delivery approaches. NVivo 11 Pro software will be used to code for thematic analysis. STATA version 15 will be used to perform quantitative analysis.

**Discussion:**

The findings of this process evaluation will provide evidence of the core active ingredients that enable the implementation of best practice nutrition intervention for people with upper gastrointestinal cancer. Elucidation of the causal pathways of successful implementation and the important relationship to contextual delivery are anticipated. With this information, a strategy for sustained implementation across broader settings will be developed which impact the quality of life and nutritional status of individuals with upper gastrointestinal cancer.

**Trial registration:**

27th January 2017 Australian and New Zealand Clinical Trial Registry (ACTRN12617000152325).

## Background

Cancers of the upper gastrointestinal tract commonly result in malnutrition [1–8]. The pathogenesis of malnutrition in this population is multifactorial. Often, one of the first symptoms of upper gastrointestinal cancer is significant weight loss which prompts individuals to seek medical attention [9, 10]. Tumour growth also impacts individuals with symptoms of pain, anorexia, dysphagia and physical obstruction resulting in concurrent reductions in oral intake [11, 12]. Commencement of oncological and radiological treatments cause further side effects that reduce oral intake including nausea, vomiting, diarrhoea, oesophagitis, mucositis and fatigue [11, 12]. The resultant malnutrition can then result in further reductions in tolerance to these treatment modalities [1, 13, 14]. Many patients therefore arrive for major surgeries with a history of malnutrition which confers risks of increased complications, longer length of stay, increased morbidity and mortality and reduced quality of life [1, 12]. Long periods of pre and post-operative fasting accompanied by prolonged periods of inadequate post-operative oral intake and impaired nutrient absorption and metabolism continue to add another dimension of risk for individual’s nutrition status to further decline [1, 7, 12, 15–19].

Recommendations of the nutrition management of individuals with upper gastrointestinal cancers has been articulated in many evidence-based guidelines and original research papers. These include: early and intensive nutrition support, weekly to fortnightly intervention, practical information and nutrition counselling about optimising nutrition intake, achieve appropriate energy and protein intakes (with the provision of nutrition support), minimising nutrition impact symptoms and the stabilisation of weight [19–24]. However, significant service gaps in the nutrition care of this patient cohort in Victoria were elucidated in a point prevalence study conducted by the Department of Health and Human Services and published in 2015 [25]. Patients with upper gastrointestinal cancer had a prevalence of malnutrition of 48%; comparative to international rates of malnutrition of 22–62% [4, 26, 27]. Almost half of these Victorian patients had not been provided with dietetic intervention [25].

We are currently undertaking a randomised controlled trial ‘Effect of early and intensive nutrition care, delivered via telephone or mobile application, on quality of life in people with upper gastrointestinal cancer’ [28]. This study is a prospective three-group intervention with a parallel economic evaluation. The three major upper gastrointestinal cancers the study concentrates its research are gastric, oesophageal and pancreatic. A pilot study conducted by our group showed benefits in participants nutrition status through early and intensive intervention [29], hence the scale up into a larger randomised controlled trial. The survival analysis of this pilot study also showed a possible survival benefit between 6 months and 1.4 years post recruitment from being exposed to this intervention [30].

Two different methods of nutrition intervention delivery will be utilised; synchronous (telephone) or asynchronous (mHealth) in addition to usual care (control) over an 18-week period. Behaviour change techniques will be used to manage nutrition impact symptoms, thus ensuring the intervention is tailored to the individual’s needs. Quality adjusted life years is the primary outcome and markers of nutritional status are secondary outcomes; all measured at baseline, three, six and 12-month time periods. The detailed protocol for the RCT has been described previously [28].

It is becoming increasingly important in translational research to not only focus on the outcomes of randomised controlled trials but to also determine the underlying processes involved in implementing an intervention [31]. Process evaluations are needed to allow for the interpretation of outcomes of trials, as causality of these outcomes can be challenging to understand [32]. This randomised controlled study is testing a complex intervention due to multiple interacting components including nutrition assessment, intervention, counselling (including the use of multiple behaviour change techniques) and then monitoring and evaluation of the effectiveness of individual patient interventions all while interacting within a multifaceted social context. The mechanisms through which interventions might bring about change in participants in this trial are essential to understand so that the potential benefits of these interventions can be replicated, particularly in clinical practice in a broad range of settings.

## Aims and objectives

This process evaluation aims to measure and compare the effectiveness of the process of intervention delivery whilst also exploring and comparing the mechanisms of action between the two intervention arms in our trial across a range of domains. The objectives/questions relating to each of these domains, and the methods of data collection and analysis are described in detail in Table 3.

1. Content
2. Dose/Contact
3. Behaviour change
4. Barriers and facilitators to engagement of participants
5. Acceptability
6. Factors mediating engagement, behaviour change and health outcomes

## Methods

### Study design

This will be a concurrent triangulation mixed methods evaluation of a randomised controlled trial of two different service delivery models of dietetic interventions in patients with oesophagogastric and pancreatic cancer. Concurrent triangulation requires that data for qualitative and quantitative analyses be collected at multiple time points, with initial analyses of qualitative and quantitative data completed separately and then combined to compare the results for interpretation [28, 33, 34]. Data related to primary outcomes will be reported separately in the main trial paper.

## Sample size calculation

**Fig. 1:**
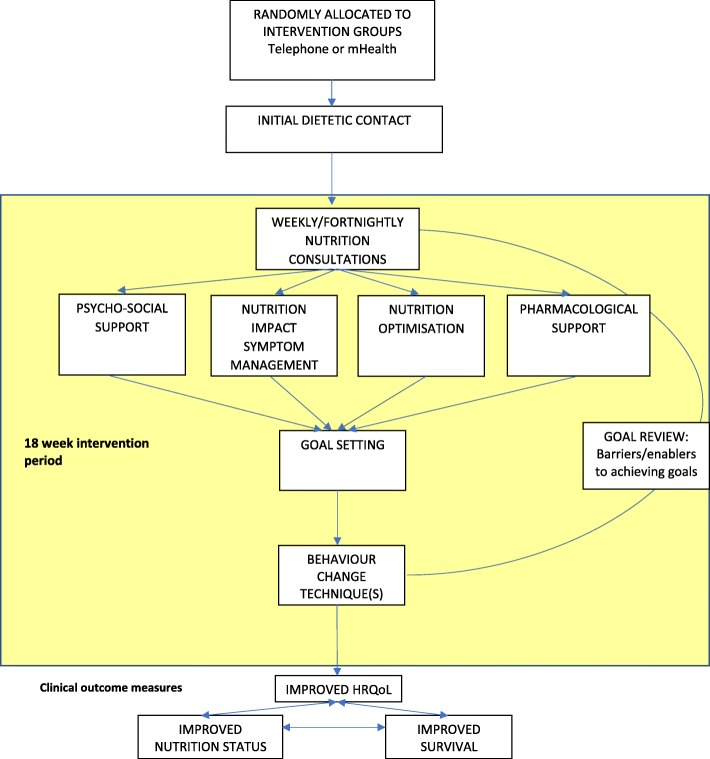
Conceptual framework. Framework of the delivery of nutrition intervention for participants who are randomised to the intervention. Dietitian completes an initial nutrition assessment over the phone. The dietitian identifies and prioritises issues to be addressed e.g. psycho-social, nutrition impact symptoms, nutrition optimisation and/or pharmacological support. Goals are set, then supporting behaviour change techniques are employed taken from the Behaviour Change Technique Taxonomy V1 (BCTTV1) [37]. At each weekly/fortnightly nutrition consultation goals are reviewed and strategies negotiated to promote achievement of goals. The effectiveness of the intervention will be assessed through the outcome measures of health-related quality of life (HRQoL). nutritional status and survival

**Fig. 2:**
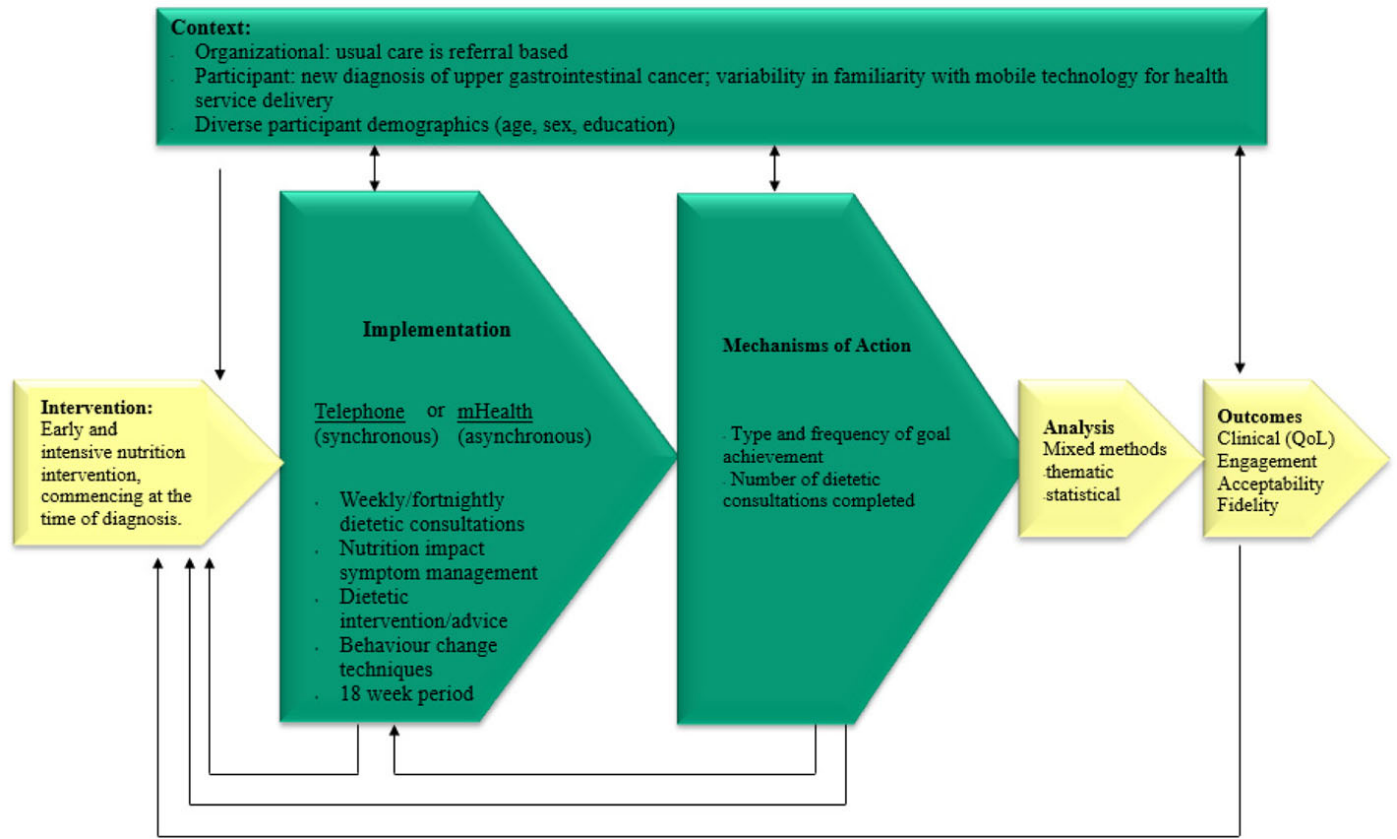
Process evaluation and mechanisms of action flow chart. This flow chart presents the key functions involved in the process and mechanisms of action evaluation of this randomised controlled trial. The green boxes form the most integral components of this evaluation. Analysis using a mixed methods approach allows for the interpretation of outcomes. Adapted from Moore et al [40]

A sample size of *n* = 33 participants per group is estimated to attain 80% power to identify a smaller standardised difference (0.70) for comparisons with the control group at the alpha = 0.05 level on the QALY lived outcome. We will therefore recruit *n* = 37 per group to account for potential drop-outs from this study (note – there were zero drop-outs for reasons other than death, from our pilot study with *n* = 21) (Figs. 1 and 2).

### Participants

Participants from both arms of the randomised controlled trial will be eligible to participate in the process evaluation. The control group participants will not participate in this evaluation. Surgeons, Oncologists, Radiation Oncologists and hospital-based dietitians will also be invited to participate.

### Intervention

**Fig. 3:**
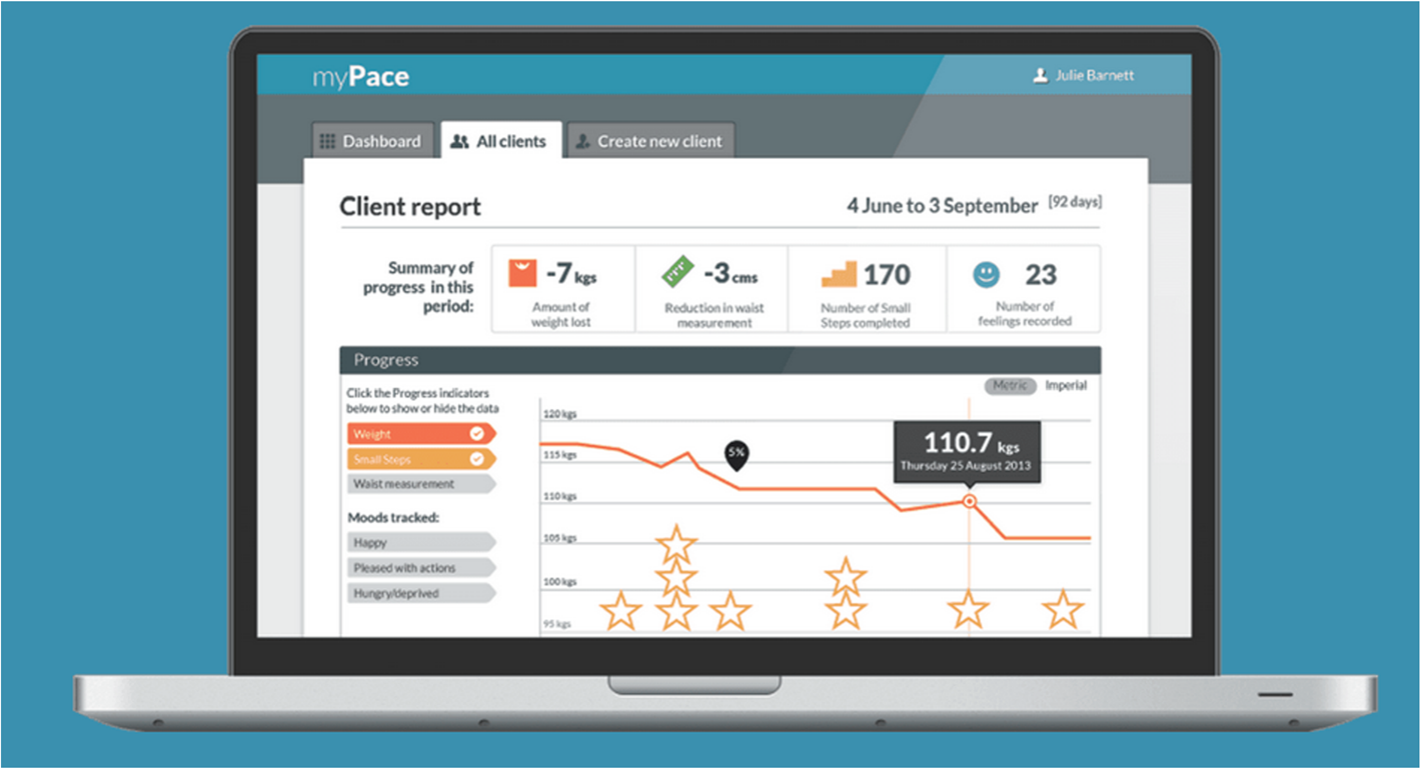
MyPace [55]

The nutrition intervention will be delivered under two conditions either via synchronous or asynchronous methods, in addition to usual care. The mHealth intervention will be delivered via MyPace, a mobile application that allows the research dietitian and participant to communicate through regular asynchronous messaging to tailor individual advice (Fig. 3). Additionally, automated daily reminders encourage self-monitoring and in combination the two techniques aim to promote sustained behaviour change [35, 36]. The telephone intervention will be delivered via a study mobile phone to participants home or mobile telephone as per their preference.

The research dietitian commences the 18-week intervention in the earliest time frame from the participant’s diagnosis, consent, recruitment and baseline data collection. An initial nutrition assessment will be carried out via telephone for both intervention groups using a standard nutrition assessment form. Throughout the intervention period, either weekly or fortnightly reviews will occur, based on the discretion of the dietitian and as per participant preference. Participants who cannot be contacted (after two attempts) via telephone, those who do not respond to messages via MyPace, or those who are in hospital at the time of review will not receive their planned review for that week. No suspension of the 18-week intervention will occur. Participants in both groups will have written information resources emailed or posted via mail. Participants requiring oral nutrition supplements (when clinically indicated) will be mailed samples and an order form will be provided via email or posted via mail.

The Behaviour Change Technique Taxonomy v1 (BCTTv1) will be used to guide the choice of behaviour change techniques that will be utilised during all participant interventions (Table 1) [37].

**Table 1:** Behaviour change techniques

Behaviour change technique	Definition [37]	Example	Classification^a^
1. Goals and Planning			
1.1 Goal setting (behaviour)	Set or agree on a goal defined in terms of the behaviour to be achieved	Set the goal of eating 5 pieces of fruit per day	Routinely Used
1.2 Problem solving	Analyse, or prompt the person to analyse, factors influencing the behaviour and generate or select strategies that include overcoming barriers and/or increasing facilitators	Prompt the patient to identify potential barriers to them drinking a particular supplement (e.g. bad taste) and discuss ways in which they could overcome them (e.g. mix with strawberries)	Supplementary
1.3 Goal setting (outcome)	Set or agree on a goal defined in terms of a positive outcome of wanted behaviour	Set a weight gain goal (e.g. 0.5 kg over 1 week) as an outcome of changed eating patterns	Supplementary
1.4 Action planning	Prompt detailed planning of performance of the behaviour (must include at least one of context, frequency, duration and intensity). Context may be environmental (physical or social) or internal (physical, emotional or cognitive)	Prompt planning the drinking of a supplement at a particular time (e.g. before work) on certain days of the week	Routinely Used
1.5 Review goal (behaviour)	Review behaviour goal(s) jointly with the person and consider modifying goal(s) or behaviour change strategy in light of achievement. This may lead to re-setting the same goal, a small change in that goal or setting a new goal instead of (or in addition to) the first, or no change.	Ask if the patient drank the supplement as planned	Routinely Used
1.6 Highlight discrepancy between current and goal (behaviour or outcome)	Draw attention to discrepancies between a person’s current behaviour (in terms of the form, frequency, duration, or intensity of that behaviour) or outcome and the person’s previously set behavioural goals or action plans	Point out that the recorded supplement intake fell short of the goal set	Routinely used
1.7 Review goal (outcome)	Review outcome goal(s) jointly with the person and consider modifying goal(s) in light of achievement. This may lead to resetting the same goal, a small change in that goal or setting a new goal instead of, or in addition to the first	Ask if the patient achieved the weight gain goal	Supplementary
2. Feedback and Monitoring			
2.1 Monitoring of behaviours by others, without feedback	Observe or record behaviour with the person’s knowledge as part of a behaviour change strategy	Have partner observe food intake behaviours and make notes on content and frequency	Supplementary
2.3 Self-monitoring of behaviour	Establish a method for the person to monitor and record their behaviour(s) as part of a behaviour change strategy	Ask the person to record daily, in a diary, the amount of food they have eaten	Supplementary
2.4 Self-monitoring of outcome of behaviour	Establish a method for the person to monitor and record the outcome(s) of their behaviour as part of a behaviour change strategy	Ask the person to weigh themselves at the end of each day, over a two-week period, and record their daily weight on a graph to increase food intake	Supplementary
2.5 Monitoring outcomes of behaviours by others, without feedback	Observe or record outcomes of behaviour with the person’s knowledge as part of the behaviour change strategy	Record weight maintenance/gain, blood glucose levels	Supplementary
2.6 Biofeedback	Provide feedback about the body using an external monitoring device as part of a behaviour change strategy	Inform the person of the blood sugar levels to improve their adoption of insulin use	Supplementary
2.7 Feedback on outcome(s) of behaviour	Monitor and provide feedback on the outcome of the performance of the behaviour	Inform the person of their stable weight following implementation of high energy, high protein diet regimen	Supplementary
3. Social Support			
3.1 Social support (unspecified)	Advise on, arrange or provide social support (e.g. from friends, relatives, colleagues,’ buddies’ or staff) or non-contingent praise or reward for performance of the behaviour. It includes encouragement and counselling, but only when it is directed at the behaviour	Arrange for a partner to encourage patient to use supplements	Supplementary
3.2 Social support (practical)	Advise on, arrange, or provide practical help (e.g. from friends, relatives, colleagues, ‘buddies’ or staff) for performance of the behaviour	Ask the partner to mix the supplement with strawberries for the patient	Supplementary
Social support (emotional)	3.3 Advise on, arrange or provide emotional social support (e.g. from friends, relatives, colleagues, buddies or staff) for performance of behaviour	Ask a patient to take a partner to your surgeon appointment	Supplementary
4. Shaping Knowledge			
4.1 Instruction on how to perform behaviour	Advise or agree on how to perform the behaviour (includes ‘Skills training’)	Demonstrate or describe to person how to prepare thickened fluids	Routinely Used
4.2 Information about antecedents	Provide information about antecedents (e.g. social and environmental situations and events, emotions, cognitions) that reliably predict performance of the behaviour	Discuss how people find it difficult to follow their diet when they attend social events	Supplementary
5. Natural consequences			
5.1 Provide information (e.g. Written, verbal, visual) about health consequences of performing the behaviour	Provide information (e.g. Written, verbal, visual) about health consequences of performing the behaviour	Present written information about the positive effect on weight and maintaining nutrition status with adoption of high energy high protein diet regimen	Supplementary
7. Associations			
7.1 Prompts / cues	Introduce or define environmental or social stimulus with the purpose of prompting or cueing the behaviour. The prompt or cue would normally occur at the time or place of performance	Put a sticker on fridge to avoid eating cheesecake	Supplementary
8. Repetition and substitution			
8.7 Graded tasks	Set easy-to-perform tasks, making them increasingly difficult, but achievable, until behaviour is performed	Ask patient to consume supplement once per day the first week, then twice per day the second week.	Supplementary
9. Comparison of outcomes			
9. 2 Consider pros and cons	Advise the person to identify and compare reasons for wanting (pros) and not wanting to (cons) change the behaviour	Advise the person to list and compare the advantages and disadvantages of drinking the supplement	Supplementary
11. Regulation			
11.1 Pharmacological Support	Provide, or encourage the use of or adherence to drugs to facilitate behaviour change	Advise the person to take regular anti-nausea medications when they are nauseated	Supplementary
12. Antecedents			
12.1 Restructuring the physical environment	Change, or advise to change the physical environment in order to facilitate performance of the wanted behaviour	Advise to make a 1 L jug of Sustagen and keep in the fridge to sip during the day	Supplementary
12.2 Restructuring the social environment	Change, or advise to change the social environment in order to facilitate performance of the wanted behaviour	Advise a person the to sit with a family member/friend at meals and snacks	Supplementary
12.6 Body changes	Alter body structure, functioning or support directly to facilitate behaviour change	Prompt use of dentures to promote food consumption	Supplementary
15. Self-belief			
15.1 Verbal persuasion about capability	Tell the person that they can successfully perform the wanted behaviour, arguing against self-doubts and asserting that they can and will succeed	Tell the person that that can successfully maintain their weight despite ongoing treatment	Supplementary
15.3 Focus on past success	Advise to think about or list previous successes in performing the behaviour	Advise to describe or list the times they were able to drink their prescribed nutrition supplements drinks during chemotherapy	Supplementary

^a^Behaviour change techniques have been classified as routinely used techniques to be used with all participants, and supplementary techniques that can be optionally be used *Adapted from BCT Taxonomy V1: 93 hierarchically-clustered techniques [37]

### Data sources

#### MyPace app

**Fig. 4:**
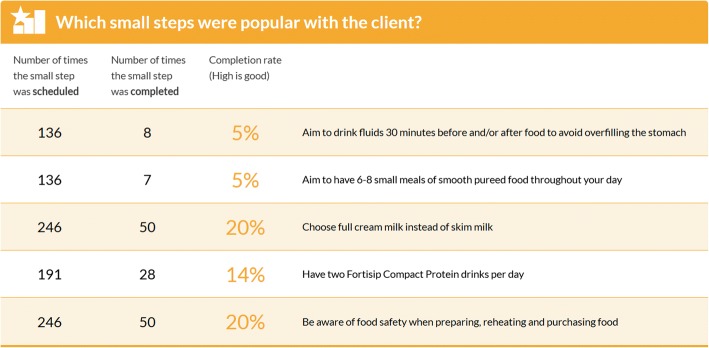
MyPace Small Steps [55]

**Fig. 5:**
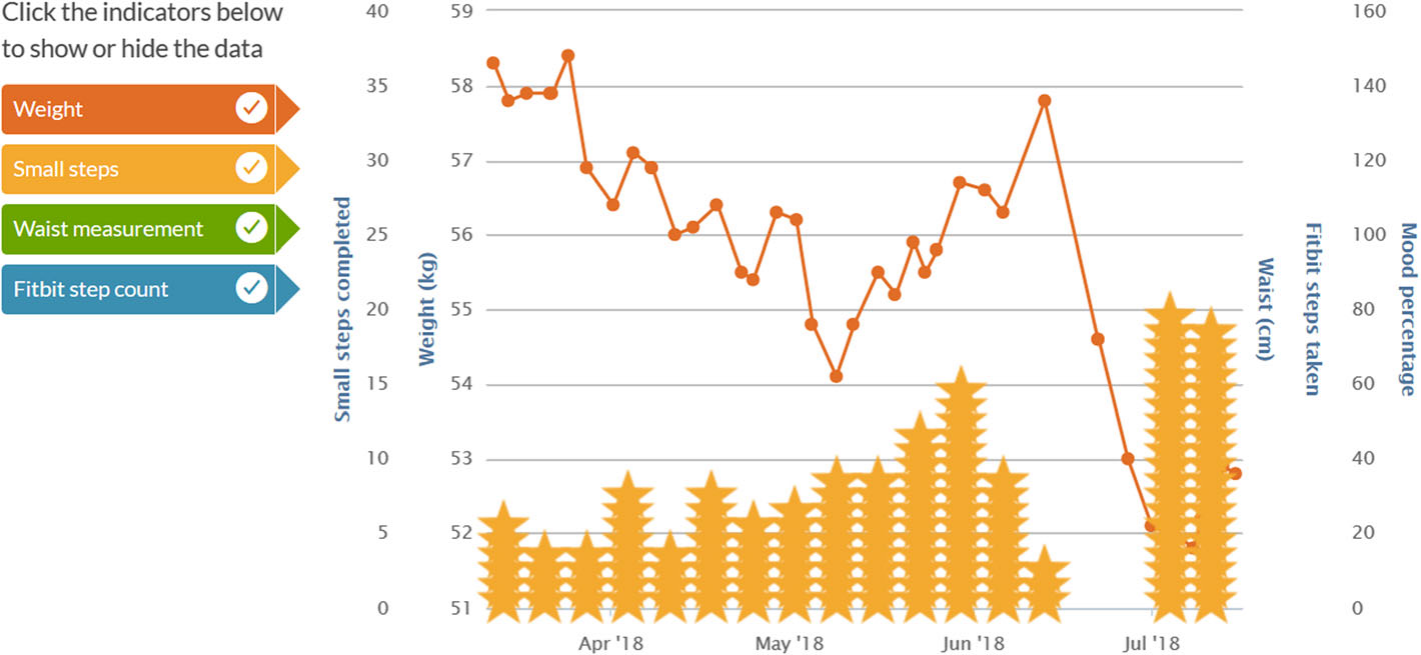
My Pace Progress Summary and Weight Graph [55]

Participants can choose to use their own home computer, mobile telephone or internet enabled tablet to download MyPace for use; alternatively, participants will be given (in person or sent via Australia Post express post) an internet enabled iPad to use for the duration of the intervention. Data from the asynchronous arm of the intervention will be collected through MyPace analytics and the initial voice recorded telephone nutrition assessment. Research dietitian directed and collaboratively set goals with the participant will be entered as Small Steps. These may be changed, ceased or added to during the intervention period. Participant completion or non-completion of Small Steps will be collected via the MyPace participant report page. This page shows the number scheduled, the number completed, and a percentage of scheduled vs completed Small Steps (Fig. 4). If a participant was lost to contact after the goal was set, it will be assumed that the goal was not achieved. Weight change throughout the intervention is recorded and graphically represented on the participants report page (Fig. 5). Weight loss/gain is summarised at the top of the report page. Participant and dietitian intervention messages will be retained.

#### Telephone recordings

Participants randomised to the telephone (synchronous) arm will have all their delivered nutrition consultations voice recorded. In addition to this, the dietitian will take written notes on a standardised nutrition initial or review form for completeness. Any additional phone calls initiated by the participant between planned consultations will also be recorded. Participants will self-report to the research dietitian at each consultation whether behaviour change goals are achieved or not achieved. During these consultations goals will also be reviewed and changed if required. If a participant was lost to contact after the goal was set, it will be assumed that the goal was not achieved.

#### Intervention delivery records

The research dietitian will maintain a set of participant records that include data on date and duration of all scheduled and non-scheduled interventions, number of attempts to contact participants (maximum of two per scheduled intervention), any missed interventions (including those who were current inpatients of a hospital) throughout the 18-week study period for both arms of the intervention. MyPace app technical issues will also be identified and recorded. All technical issues with MyPace will be addressed through telephone calls to participants that focus only on the resolution of these issues. These issues will also be escalated to software support services.

A record of the written nutrition education materials that patients require to address nutrition support needs and symptom management, any oral nutrition support products sent to participants to trial and order forms for home delivery of the oral nutrition support products. The delivery method whether email or mailed via Australia Post express post will be recorded.

Topics for goal setting and the behaviour change techniques used to achieve these goals will be recorded from the dietetic consultations (i.e. telephone calls, MyPace messaging and MyPace Small Steps). These will include:

##### Nutrition optimisation

The research dietitian will conduct a full initial nutrition assessment of each participant at their initial contact. The participant’s nutrition needs are then identified and prioritised**,** e.g. a deficit in energy and protein requirements which needs to be addressed for weight stability during treatment. The dietitian and participant will then set goals for addressing these nutrient deficits. Behaviour change techniques are used to support goal setting e.g. problem solving and action planning. At each participant review their goal achievement is assessed; goals can be ceased, modified or continued in addition to the setting of new goals.

##### Nutrition impact symptom(s) management

Nutrition impact symptoms including anorexia, early satiety, dysphagia, reflux, regurgitation, odynophagia, xerostomia, mucositis, oesophagitis, gastritis/enteritis, hiccups, radiation pneumonitis, oesophageal stricture/stenosis, nausea, vomiting, fatigue, neutropaenia, constipation, diarrhoea, steatorrhoea (pancreatic exocrine insufficiency), diarrhoea and cholinergic syndrome, bloating, pain and laryngo-pharyngeal dysaesthesia will be recorded for each participant, and strategies to assist with the management of these will occur through goal setting.

##### Pharmacological support

Pharmacological support relates to the recommendations for the use of and adherence to medications (in line with the treating medical team recommendations as per patient self-report) to assist with the management of nutrition impact symptoms; examples include antiemetics, aperients and pancreatic enzyme replacement therapy.

##### Psycho-social support

Sourcing psychological support will include recommendations to seek out support through family/friends, social workers or psycho-oncology clinics through the hospital the participant is affiliated with, the Cancer Council telephone information and support service, and Lifeline.

### Semi-structured post intervention interviews

At the conclusion of the 18-week intervention, all participants will be invited by an independent researcher to participate in semi-structured post intervention interviews. Participants choosing to participate may nominate a family member or carer to complete the interview on their behalf. All interviews are facilitated by a researcher (KH) with knowledge of the RCT but who was not involved with the delivery of the nutrition intervention. Interviews will be conducted via telephone and voice recorded. The goal of the interviews is to gather data from the perspective of the participants on 1) the relevance of the intervention to their needs, 2) the flow of communication with the dietitian, 3) the motivation for taking part in the study, and 4) promoting self-management. Modifications to the interview schedule will occur through an iterative/reflective process during data collection to capture the participants’ experiences during the intervention (Table 2).

**Table 2:** Sample Questions of the post intervention semi-structured interviews with participants

Questions	Logic
As someone who has cancer, what is it like for you managing your nutrition?	Living with cancer
Tell me about the experience you had as a participant in this study. - Did it meet your nutritional needs?	Relevance to the patient
What was it like for you being contacted by the dietitian frequently? - Tell me what was challenging? - Would you change anything (throw something out, add something in?) - What did you like? - (iPad group) what was it like learning a new App?	Self-management practice
Tell me what is was like communicating with a health profession using the phone (or iPad)? - What helped or hindered communication between you and the dietitian? - What would have made this experience better for you? - Describe any challenges you had communicating with the dietitian - What do you need to facilitate communication? - What could we have improved the way we delivered the nutrition to you?	Communication
If you could design this service, what would be the key features of the service? - Tell me about the scheduling of the consultations - How important is flexibility? - What could we have done to support you better?	Unmet care needs
What motivated you to take part in this study?	Motivation
Is there anything else you’d like to tell me about that relates to your experience throughout the intervention?	Overall experience
What role did your family play in your nutrition care during the study period?	Social influences
Did you contact the dietitian as often as you wanted to? - What motivated or stopped you from using the app?	Contact
Did you have any problems using the app or contacting the dietitian? - If you had problems, what were they? - How did you solve the problem?	Technical Problems
Did any of your family members help you with the app or dietetic consultations? - How important was it to you to be able to involve someone else in this service? - Would they like to share with me their experience of the intervention?	Family/Carer engagement

### Semi-structured health professional interviews

At the conclusion of the study, the multidisciplinary treating team including Surgeons, Oncologists, Radiation Oncologists and hospital-based dietitians will be invited by an independent researcher to participate in semi-structured interviews. All interviews are facilitated by a researcher with knowledge of the RCT but who was not involved with the delivery of the nutrition intervention. Interviews are conducted either in person or via telephone and voice recorded. The goal of the interviews is to gather data from the perspective of the health professionals involved in treating the participants and explore acceptability of the model of nutrition intervention delivery.

### Participant data at baseline and 6 months

Demographic data including gender and age, anthropometrical data on self-reported body weight, change in HRQoL (scores from the EQ-5D-5 L instrument) [38] and survival for participants in the two arms of the intervention will be collected by an independent blinded researcher at baseline and 6 months. Survival data may be ascertained through a family member at the time of routine contact at follow-up assessments where possible. The Victorian Cancer Registry, which receives death notifications from the Registrar of the Victorian Department of Births Deaths and Marriages for all people who die from cancer in Victoria, will then be used to confirm mortality data.

Prior and/or regular use and confidence with technology will be assessed by five questions during baseline data collection; 1) Do you use email? 2) Do you own a smartphone? 3) Do you own an iPad (or internet enabled tablet)? 4) Do you feel confident to communicate with a health professional using messages from your smartphone or iPad? 5) Do you regularly (at least once per day) use your smartphone for something other than for making calls?

### Data analysis

Classification of research dietitian directed goals, collaboratively set goals, nutrition impact symptoms and behaviour change techniques used by the dietitian will be undertaken by content analysis of audio files, MyPace messages and setting of Small Steps between the research dietitian and participants. A classification matrix will be used to facilitate this process.

Time from baseline assessment completion until research dietitian attempt to contact and to first successful contact with participant will be compared between the two intervention groups using Cox proportional hazards regression analysis. Descriptive statistics will be used to characterise the methods by which participants initiated contact with the research dietitian. Frequency of contact with the research dietitian and participants will be compared between groups using either Poisson or negative binomial regression depending on the data distribution. The proportion of participants who had at least fortnightly contact, who were sent written nutrition education materials, and a trial pack of oral nutrition support products will be compared using logistic regression.

The number of behaviour change goals per participant will be compared between groups using Poisson or negative binomial regression based on the data distribution. The base analysis will compare the number of goals achieved in the telephone group based on self-report during synchronous reviews to the number of small step goals completed for the asynchronous group. A sensitivity analysis will be conducted for the asynchronous group where goals reported as being completed in written message correspondence but not in the Small Steps will additionally be reported as achieved goals along with Small Steps.

A thematic analysis of audio recordings of both the synchronous interventions and post-intervention semi-structured interviews and MyPace messages will allow for the identification, description and contrast of the types of barriers and facilitators to engagement of participants between intervention groups.

A thematic analysis of audio recordings of the post study health professional interviews will identify and describe the acceptability of both models of nutrition intervention from the point of view of the treating hospital team.

We will build a multiple regression model (Poisson or negative binomial regression dependent on the data distribution) that predicts the number of sessions completed by the participant with the research dietitian. We will use the model building approach described by Hosmer and Lemeshow [39] .

To explore the relationships between participant demographics, intervention focus (examples include nutrient intake, nutrition impact symptoms), dose, timing, and frequency and whether the goal set was achieved, we will build a multiple logistic regression model using the model driven approach described by Hosmer and Lemeshow [39].

To explore the relationships between participant demographics, engagement and goal achievement with health outcomes, we will build separate models for the three health outcomes of: 1) weight being within +/− 5% of baseline weight at 6 months analysed via multiple logistic regression, 2) HRQoL at 6 months analysed via multiple regression analysis and 3) survival at 12 months analysed using multiple Cox proportional hazards analysis. We selected the outcome of weight being within +/− 5% based on previous literature demonstrating weight stability during cancer treatment as an important goal to achieve [3, 20, 21] (Table 3).

**Table 3:** Process evaluation objectives, questions and collection of data

Domain	Questions	Data source	Data Analysis Technique
1). Content	• What are the number of instructions provided by the dietitian per participants? • What are the content areas of instructions/goals provided by the research dietitian to participants? To measure and compare: • Classification of the research dietitian directed goals (where the dietitian has directed the focus, dose, frequency and timing of the goal) • Classifications of the collaboratively set goals (where the participant has decided upon either the focus, dose, frequency or timing of the goal) • Classifications of the nutrition impact symptoms reported by the participant to the research dietitian • Classifications of the behaviour change techniques used by the research dietitian to support achievement of the goal • Classification of the written nutrition education materials provided by the research dietitian to the participant	Audio recordings, MyPace messages and emails, paper based initial nutrition assessment and review forms	Content analysis using a classification matrix
2). Dose/Contact	To measure and compare: • Time from baseline assessment completion until research dietitian attempt to contact the participant to commence intervention • Time from baseline assessment completion until first successful contact between research dietitian and participant • The methods used by participants to initiate contact with dietitian • The frequency of contact between research dietitian and participants • The proportion of participants who had at least one contact with the research dietitian every 2 weeks for the duration of the 18-week intervention period • The proportion of participants who were sent written nutrition education materials • The proportion of participants who were sent a trial pack of oral nutrition support products • The proportion of participants who have family member/carer involvement in intervention interactions	Audio recordings, MyPace messages and emails, paper based initial nutrition assessment and review forms, MyPace analytics, post intervention semi-structured interviews	Descriptive statistics, Cox proportional hazards regression analysis, either Poisson or negative binomial regression (depending on the data distribution), logistic regression
3). Behaviour change	• To measure and compare the number of behaviour change goals achieved per participant between groups.	Audio recordings, MyPace messages and emails, paper based initial nutrition assessment and review forms	Poisson or negative binomial regression (based on the data distribution), sensitivity analysis
4). Barriers and facilitators to engagement	• Identify, describe and contrast the types of barriers and facilitators to engagement of participants between the intervention groups	Audio recordings, MyPace messages and emails, research dietitian field notes, semi-structured post intervention participant interviews	Thematic analysis
5). Acceptability	• The acceptability of the intervention from the perspective of participants and research dietitian and treating team including Surgeons, Oncologists/Radiation Oncologists, hospital-based dietitians)	Semi-structured post study health professional interviews	Thematic analysis
6). Factors mediating engagement, behaviour change and health outcomes	• What are the inter-relationships between a range of demographic, process, behaviour change and health outcome measures to better understand the potential mechanisms of action of the interventions delivered? • What are the relationships between participant demographics and measures of engagement.	Baseline demographic and technology confidence data, weight and HRQoL data at baseline and 6 months, data on survival at 6 months, intervention delivery records, research dietitian field notes, semi-structured post intervention participant interviews	Poisson or negative binomial regression (dependent on the data distribution), multiple logistic regression, multiple regression analysis and multiple Cox proportional hazards analysis

*Adapted from [32, 56, 57]

## Discussion

The concurrent, novel three group randomised controlled trial will provide evidence as to whether early, intensive nutrition intervention, delivered via mHealth or telephone leads to better health outcomes in patients with upper gastrointestinal cancer along with evidence regarding the most cost-effective and acceptable delivery method of this intervention. Evaluations of complex interventions using both qualitative and quantitative evidence allow for the multiple interacting components of a study to be fully elucidated and well understood [40, 41]. The purpose of this study is to outline the comprehensive process evaluation that will be used to provide the information required to interpret the clinical outcomes of this trial; to determine the mechanisms of action and the complex interacting contextual factors in which this action occurs. This will allow the drawing of links between the processes of the intervention delivered with participants (family/carer) engagement, health related behaviour change and overall health outcomes.

Telephone and web-based delivered interventions have been lauded as important approaches to provide convenient, accessible, equitable and cost effective health behaviour change interventions that also encompass personalised, individually tailored information [42–44]. However, it is unclear which method, in the population of patients undergoing treatment for upper gastrointestinal cancer, is more effective and acceptable in changing health behaviours, improving health outcomes and ultimately survival. There are few studies that explore the delivery of health behaviour change interventions by ehealth methods during cancer treatment, most studies focus on prevention and survivorship interventions [45–48].

Patient engagement in behaviour change interventions is paramount to their success. The definition of engagement in the literature has lacked consistency, therefore its measurement has been heterogenous and challenging to compare. This is particularly evident in digital behaviour change interventions where usage metrics have been used as typical markers of engagement [49, 50]. However, engagement is also significantly affected by a number of variables including patient demographics and individual circumstances; care setting, cancer type, stage and treatment modality(ies), technology acceptance, confidence and functions used and family/carer support [50]. This evaluation will not only measure objective data analytics but subjective qualitative analysis. This allows for the measurement of the initiation of engagement but also sustained engagement over the life of the 18 week intervention time frame. The qualitative thematic analysis will also incorporate the barriers and facilitators to participant engagement. The differences between the two delivery methods will be highlighted, allowing the determination of whether intervention group assignment also effects participant engagement.

Behaviour change techniques used within complex behaviour change interventions are imperative components that are essential to report to allow replicability [51–53]. Literature supports studies that use a variety of behaviour change techniques in producing larger outcome effects [54]. Our study encompasses a range of five routinely used and twenty-three supplementary behaviour change techniques used in combination to elicit health behaviour change. The evaluation of the use of these techniques will allow the clarification of which specific techniques used in combination elicited the most effective behaviour change in participants.

The mixed method approach is a strength of this evaluation, allowing both qualitative and quantitative data collection and analysis. This will provide a much broader and encompassing insight into the implementation of the interventions; the acceptability of patients (families/carers) and health professionals and the complex mediators at play. Self-reported semi-quantitative post intervention interviews may introduce reporting biases due to death, uncontactable participants or those declining to participate. Of those who do participate, the interviews will be conducted retrospectively so may add recall bias and responding in a desirable manner. This also adds to the burden of research which may decrease respondent’s likelihood to participate.

## Conclusion

Early delivery of nutrition care to people diagnosed with upper gastrointestinal cancer is best-practice. Often, health care services have limited resources and therefore need to prioritise based on a balance between cost and benefit. By undertaking a process evaluation that elucidates mechanisms of action, there should be greater success in moving from the research setting to the real-world setting. There will be a greater understanding of the active ingredients required for successful delivery of early and intensive nutrition care via the telephone or mHealth. This evidence can then be translated, integrated and scaled up into a delivery approach to reach large numbers of patients through a state-wide hub.

## Abbreviations

eHealth: Electronic Health
HRQoL: Health Related Quality of Life
mHealth: Mobile Health
